# Recent Progress in Nitro-Promoted Direct Functionalization of Pyridones and Quinolones

**DOI:** 10.3390/molecules25030673

**Published:** 2020-02-05

**Authors:** Feiyue Hao, Nagatoshi Nishiwaki

**Affiliations:** 1 School of Pharmaceutical and Materials Engineering, Taizhou University, Jiaojiang 318000, China; haofeiyue@tzc.edu.cn; 2 School of Environmental Science and Engineering, Kochi University of Technology, Tosayamada, Kami, Kochi 782-8502, Japan

**Keywords:** nitro, pyridone, 1-methyl-2-quinolone, cycloaddition, direct functionalization

## Abstract

Nitro group is one of the most important functional groups in organic syntheses because its strongly electron-withdrawing ability activates the scaffold, facilitating the reaction with nucleophilic reagents or the Diels–Alder reaction. In this review, recent progress in the nitro-promoted direct functionalization of pyridones and quinolones is highlighted to complement previous reviews.

## 1. Introduction

**Figure 1:**
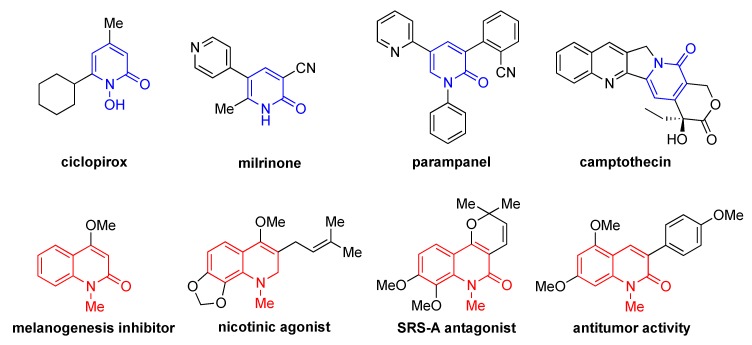
Biological activities of pyridones and quinolones.

Natural and synthetic aza-heterocycles represent an important class of organic compounds [1,2,3,4,5]. Among the large number of aza-heterocycles available, pyridones and quinolones, both of which have a common six-membered aza-framework, exhibit a wide range of pharmacologically important activities (Figure 1) [6,7,8,9,10]. Therefore, various methods for the preparation of structurally diverse pyridones and quinolones have been studied in detail [6,11,12,13,14,15,16,17,18,19,20,21].

**Figure 2:**
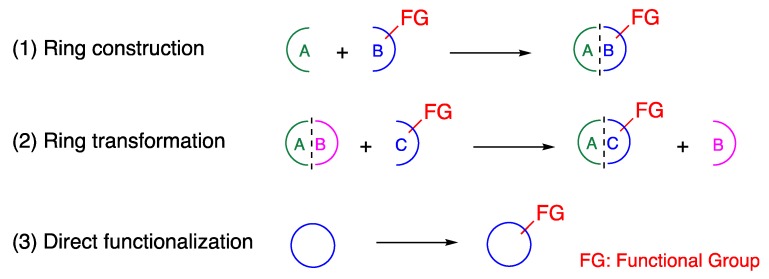
Conventional strategies for the functionalization of aza-heterocycles.

Conventional strategies for the synthesis of aza-heterocycles involve (1) construction of aza-heterocycle frameworks from prefunctionalized starting materials, (2) ring transformation leading to aza-heterocycle frameworks, and (3) direct functionalization of aza-heterocycle frameworks, which are supplementary to each other (Figure 2) [22].

**Figure 3:**
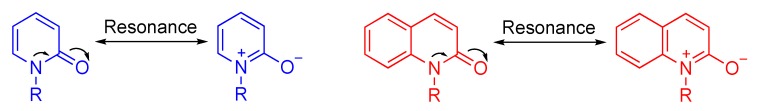
Resonance structure of pyridone framework.

Among these three protocols, direct functionalization of aza-heterocycles, for preparing new diversely functionalized heterocycles, is the most efficient approach from a practical viewpoint, because it requires only simple experimental manipulations. Accordingly, the development of easy and efficient methods for the direct functionalization of quinolone and pyridone frameworks is highly demanded. However, only a few such methods are currently available because these scaffolds are inert due to the aromaticity (Figure 3) [22].

To the best of our knowledge, the currently used methods for direct functionalization of the quinolone and pyridone scaffolds are mainly focused on transition-metal-catalyzed cross-coupling and C–H activation reactions [6,11,12,13,14,15,16,17,18,19,20,21]. However, most of these methods suffer from some limitations, such as the use of potentially poisonous and expensive noble metals, along with harsh reaction conditions.

**Scheme 1:**
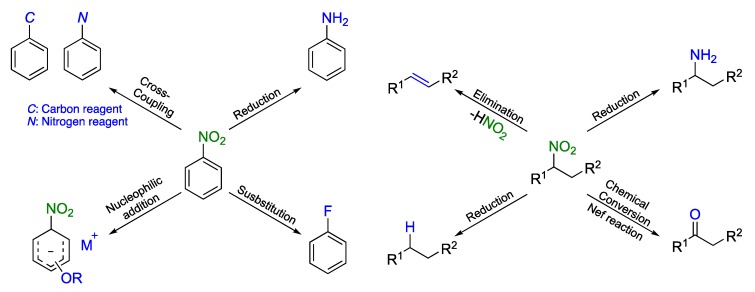
Properties of a nitro group.

However, the nitro group, which is often described as a “synthetic chameleon [23],” serves as a precursor for versatile functionalities, such as formyl, acyl, cyano, and amino groups (Scheme 1) [24,25,26,27,28]. Moreover, the nitro group has been proved to activate many different scaffolds because of its strong electron-withdrawing ability, facilitating the reaction with nucleophilic reagents [29,30]. The nitro group is also a good leaving group, which is often involved in addition–elimination reactions [31,32].

Based on these significant properties of the nitro group, the synthetic utility of nitrated aza-heterocycles in the preparation of functionalized aza-heterocycles has been widely investigated [33]. However, electrophilic nitration of pyridines and quinolines is difficult because of the electron deficiency of the aromatic cores. On the contrary, it is possible to nitrate pyridones and quinolones because the dearomatization of these scaffolds is easier than that of pyridines and quinolines. Indeed, the introduced nitro groups activate the scaffolds to facilitate direct functionalization, which affords structurally diverse aza-heterocycles. Herein, recent progress in the nitro-promoted direct functionalization of pyridones and quinolones in the past couple decades is highlighted.

## 2. Cycloaddition of Nitropyridones

The nitro group is a strongly electron-withdrawing group that reduces the electron density on the scaffold. Further, 2-pyridones possessing a nitro group are highly electron-deficient, and they serve as dienophiles that undergo Diels–Alder (D–A) cycloaddition with electron-rich dienes, forming fused aza-heterocycles [34].

When 5-nitro-2-pyridones **1** are reacted with 2,3-dimethyl-1,3-butadiene **2**, quinolones **3** are formed via regioselective D–A cycloaddition at the 5- and 6-positions and subsequent aromatization accompanied by elimination of nitrous acid (Table 1). For 5-nitropyridone bearing a methoxycarbonyl group at the 3-position, the D–A reaction occurs chemoselectively to yield the corresponding 3-functionalized quinolone **3c**.

**Scheme 2:**
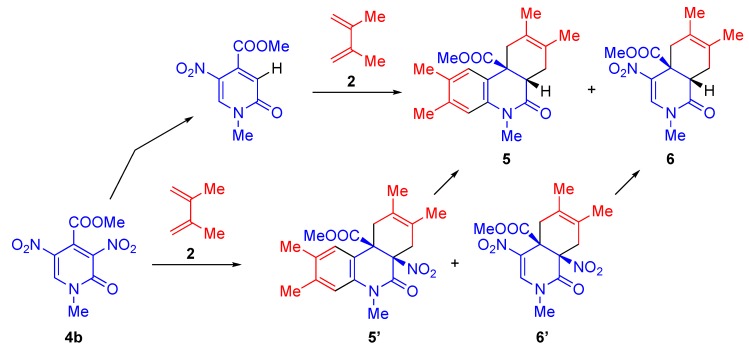
Two plausible pathways for cycloadducts **5** and **6** including denitration.

It is known that 5-nitropyridones **4** possessing electron-withdrawing groups at the 3- and/or 4-positions have two electron-deficient sites on the ring. When these substrates are subjected to D–A reactions with diene **2**, the reaction proceeds stereoselectively to produce the functionalized *cis*-adducts **5** and **6**, accompanied by denitration (Table 2). Since the reaction is conducted under harsh conditions, the denitration of either pyridone **4b** or the cycloadducts **5′** and **6′** might occur (Scheme 2), however a detailed explanation has not been reported in the literature [34].

D–A cycloaddition of 1-unsubstituted 3-nitro-2-pyridones **7a** with diene **2** yields the *cis*-condensed tetrahydroisoquinolone **8a** stereoselectively. For 1-methyl-3-nitro-2-pyridone **7b**, *cis*-tetrahydroisoquinolone **8b** as well as aromatized isoquinolone **9b** is formed via dehydrogenation and release of a nitrous acid. The use of a substrate with 4-methoxycarbonyl substitution affords *cis*-tetrahydroisoquinolone **8c** as the sole product (Table 3).

The reaction of 1-unsubstituted 3,5-dinitropyridone **10a** gives an aromatized isoquinolone **11a** via cycloaddition at the 3- and 4-positions, followed by dehydrogenation and elimination of nitrous acid; an aromatized phenanthridone **12a** is also obtained via double D–A adduct formation (Table 4). However, the reaction of 1-methyl-3,5-dinitro-2-pyridone **10b** furnishes not only 4-nitroisoquinolone **11b** and phenanthridone **12b**, but also *cis*-tetrahydroisoquinolone **8b**, via cycloaddition at the 3- and 4-positions accompanied by heating-promoted elimination of the nitro group at the 5-position. D–A reactions of 3-nitro-2-pyridones **10c** and **10d** with 5-methoxycarbonyl substitution mainly yield the aromatized isoquinolones **11c** and **11d**, respectively, in addition to the incompletely aromatized *cis*-phenanthridone adducts **13c** and **13d**, respectively.

## 3. Cycloaddition of Nitroquinolones

The D–A reactions at the nitroalkene moiety of 3-nitrated 1-methyl-2-quinolones **14** with electron-rich dienes yield aromatized phenanthridone derivatives **15** (Table 5). Although this method enables simultaneous C–C bond formation at the 3- and 4-positions of the quinolone framework, harsh reaction conditions must be employed [35,36].

**Scheme 3:**
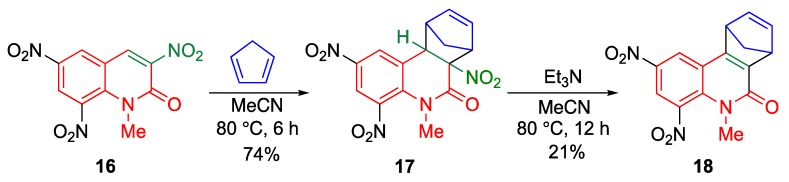
Diels-Alder cycloaddition of trinitroquinolone **16** with cyclopentadiene.

**Scheme 4:**
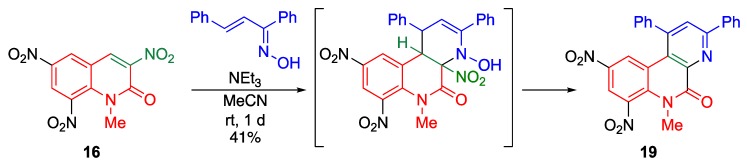
Cycloaddition of **16** with α,β-unsaturated oxime.

On the contrary, 1-methyl-3,6,8-trinitro-2-quinolone **16** undergoes cycloaddition with dienes easily under mild conditions (Scheme 3). Indeed, the cycloaddition of **16** with cyclopentadiene proceeds smoothly to furnish a tetracyclic compound **17** that aromatizes via elimination of a nitrous acid in the presence of triethylamine to afford compound **18** [37]. Similarly, the cycloaddition using α,β-unsaturated oxime, instead of cyclopentadiene, as a heterodiene affords the polycyclic diazaphenanthrene **19** (Scheme 4) [38].

**Figure 4:**
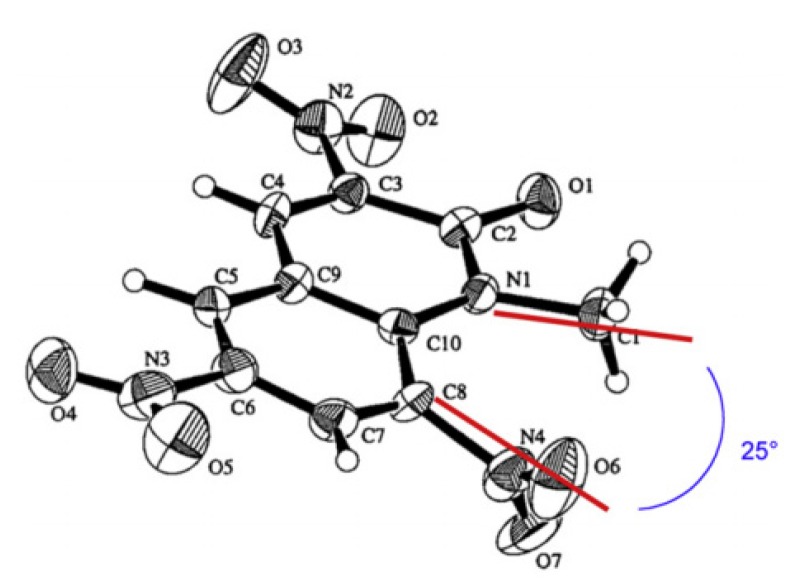
ORTEP (30% probability ellipsoids) view of trinitroquinolone **16**.

The high reactivity of trinitroquinolone **16** is due to the steric repulsion between the 1-methyl and 8-nitro groups, disturbing the coplanarity of the pyridone moiety and the benzene ring. Consequently, the pyridone ring of **16** loses its aromaticity and serves as an activated nitroalkene (Figure 4) [39].

**Figure 5:**
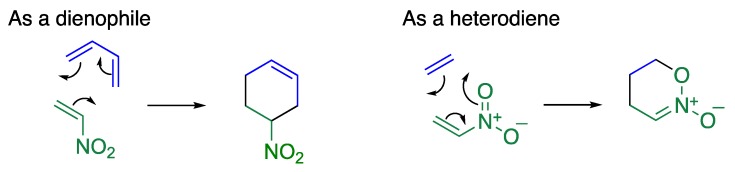
Dual behaviors of a nitroalkene in the cycloaddition reaction.

**Scheme 5:**
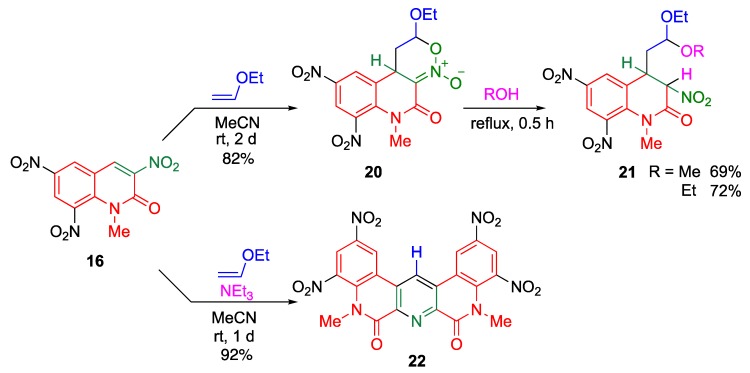
Cycloaddition of **16** with ethoxyethane.

A nitroalkene shows dual behavior in cycloaddition reactions (Figure 5). In reaction with a diene, the nitroalkene serves as a dienophile to form a cyclohexene ring. On the other hand, it serves as a heterodiene in reaction with an electron-rich alkene to construct an oxazine ring. The nitroalkene moiety of trinitroquinolone **16** also serves as a heterodiene in the reaction with ethoxyethene to construct a fused oxazine ring **20** (Scheme 5) [38], which yields an acetal **21** via ring-opening reaction upon treatment with alcohol under reflux conditions.

**Scheme 6:**
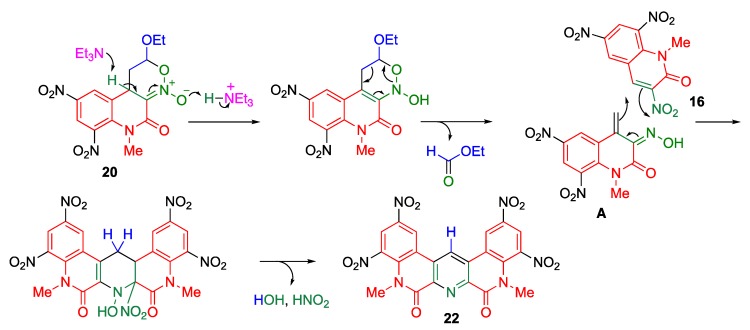
A plausible mechanism for the formation of product **22**.

Interestingly, a quinolino[3, 4-*b*][1,9]diazaphenanthrene derivative **22** is formed when the same reaction is conducted in the presence of triethylamine (Scheme 5) [38]. A plausible mechanism is shown in Scheme 6. After forming the cyclic nitronate **20**, triethylamine assists the proton transfer from the 4-position to the anionic oxygen of the nitronate. The subsequent retro D–A reaction gives the α,β-unsaturated oxime **A**, accompanied by a loss of ethyl formate. Oxime **A** serves as an electron-rich heterodiene that undergoes cycloaddition with another molecule of **16** to afford a new pyridine ring, and subsequent aromatization and elimination of nitrous acid and water furnishes the polycyclic product **22**. In this reaction, two molecules of trinitroquinolone **16** undergo two kinds of cycloaddition reactions: one molecule serves as a heterodiene and the other serves as a dienophile. This is the first example of a nitroalkene that exhibits dual behavior in the same reaction mixture (Figure 5).

## 4. Nitro-Promoted Cyclization of Pyridones via Nucleophilic Addition

**Scheme 7:**
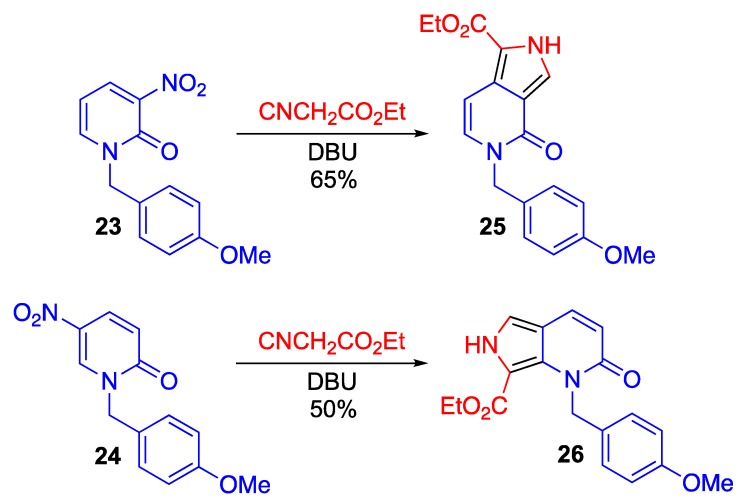
Cyclization of nitropyridones **23** and **24**.

The strongly electron-withdrawing ability of the nitro group activates the scaffold for nucleophilic attack at the vicinal position on the nitroalkene. The nitroalkene moiety of nitropyridones is also susceptible to nucleophilic reaction. Indeed, 1-substituted nitropyridones **23** and **24** react with ethyl isocyanoacetate in the presence of 1,8-diazabicyclo[5.4.0]undec-7-ene (DBU) to afford the pyrrolopyridine derivatives **25** and **26**, respectively (Scheme 7) [40]. In the latter case, nucleophilic attack of isocyanoacetate occurs regioselectively at the 6-position.

**Scheme 8:**
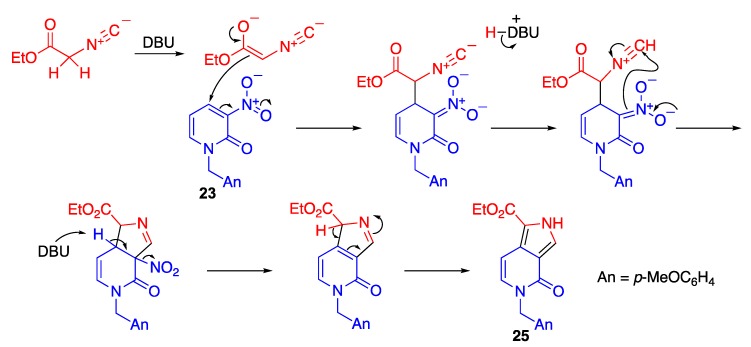
A plausible mechanism for cyclization of nitropyridone **23** with isocyanoacetate.

The reaction is initiated by the nucleophilic addition of isocyanoacetate to nitropyridone under basic conditions to produce an anionic intermediate stabilized by the nitro group (Scheme 8). Then, the nucleophilic attack of the nitronate to the protonated isocyano group affords dihydro-2*H*-pyrrole, from which a pyrrole ring is produced via aromatization by elimination of nitrous acid.

## 5. Nitro-Promoted Direct Functionalization of Quinolones

### 5.1. Direct C–C Bond Formation at the 4-Position via Cine-Substitution

**Scheme 9:**
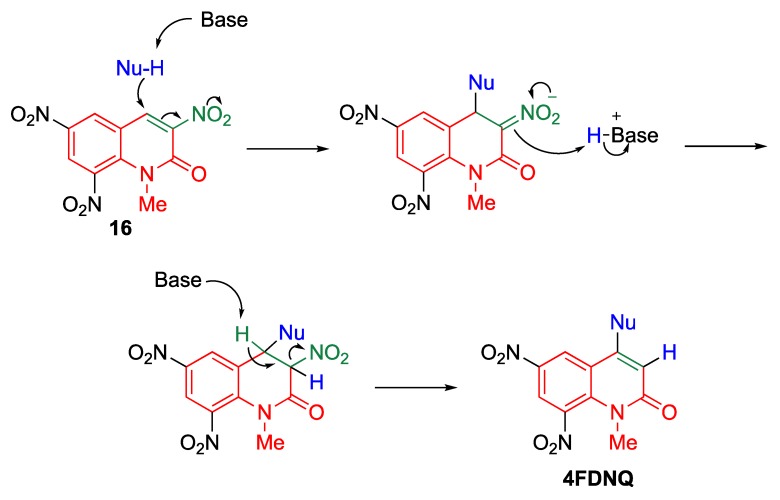
*cine*-Substitution of trinitroquinolone **16**.

To the best of our knowledge, the currently used methods for direct C–C bond formation in 1-methyl-2-quinolone (**MeQone**) framework are mainly limited to transition-metal-catalyzed cross-coupling or C–H activation reactions [11,12,13,14,15,16]. As an alternative, the introduction of a nitro group has proved helpful in facilitating direct functionalization of the **MeQone** framework, affording diversely functionalized **MeQone**s. Indeed, *cine*-substitution of trinitroquinolone **16** with various nucleophiles can easily proceed to afford 4-functionalized 6,8-dinitro-1-methyl-2-quinolones (**4FDNQ**) [22]. Initially, the nucleophilic substitution proceeds at the 4-position of **16** to form an adduct intermediate; then, a proton is transferred from the basic group to the 3-position of the adduct intermediate, affording 3,4-dihydroquinolone. The subsequent elimination of nitrous acid, accompanied by aromatization, yields **4FDNQ** (Scheme 9). This reaction enables regioselective functionalization at the 4-position of the **MeQone** framework. Direct C–C bond formation at the 4-position of the **MeQone** framework is easily achieved upon treatment of **16** with carbon nucleophiles, including 1,3-dicarbonyl compounds, nitroalkanes, aldehydes/ketones, enamines, cyanides, and phenoxides, leading to the formation of versatile skeletons.

#### 5.1.1. *cine*-Substitution of Trinitroquinolone with 1,3-dicarbonyl Compounds

When trinitroquinolone **16** is reacted with 1,3-dicarbonyl compounds in the presence of triethylamine, 4-position functionalization is efficiently achieved via *cine*-substitution (Table 6) [41]. Diketones, keto esters, and diesters can be used as nucleophiles in this reaction to afford the corresponding products **27a**–**e**.

When the nitro group at the 8-position is removed, no reaction occurs, even under heating. On the other hand, *cine*-substitution proceeds smoothly even upon replacement of the electron-withdrawing nitro group of **16** with an electron-donating methyl group (Table 7). These results indicate that the steric repulsion of this substituent with the 1-methyl group activates the **MeQone** framework, as mentioned in Section 3 [42].

#### 5.1.2. *cine*-Substitution of Trinitroquinolone with Nitroalkanes

Nitroalkylation of trinitroquinolone **16** is also achieved by using a nitroalkane as a carbon nucleophile in the presence of triethylamine (Table 8) [43]. While primary nitroalkanes undergo *cine*-substitution efficiently at room temperature, secondary nitroalkanes with steric hindrance are less reactive, requiring longer reaction times and affording relatively low yields.

#### 5.1.3. *cine*-Substitution of Trinitroquinolone with Aldehyde, Ketones and Enamines

Besides aldehydes, functionalized ketones, such as aliphatic, alicyclic, aromatic, and heteroaromatic ketones work well as carbon nucleophiles in the *cine*-substitution of trinitroquinolone **16**, giving acylmethylated products (Table 9) [44]. Since the acylmethyl group can serve as a scaffold for further chemical transformations, this method can be useful for the construction of a new library of compounds with **MeQone** framework.

More-reactive enamines can also be used as nucleophiles instead of ketones, which undergo *cine*-substitution in the presence of water at room temperature. After the addition of enamine to trinitroquinolone **16**, hydrolysis of the formed iminium ion forms an acylmethyl group. In this case, the product is obtained as a morpholinium salt **30** (Table 10) [44].

#### 5.1.4. *cine*-Substitution of Trinitroquinolone with Phenoxides

**Figure 6:**
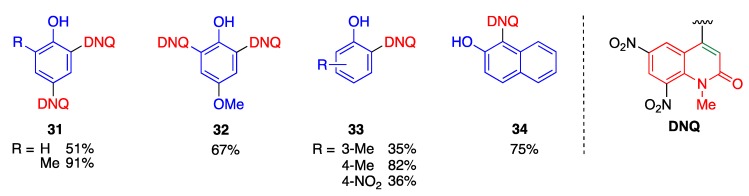
*cine*-Substituted products from **16** and potassium phenoxides.

A combination of electrophilic trinitroquinolone **16** and nucleophilic phenoxide ions results in direct arylation of the **MeQone** framework (Figure 6) [45]. When **16** is treated with potassium phenoxides possessing electron-donating groups, double *cine*-substitution proceeds to afford bis(quinolyl)phenols **31** and **32**. On the other hand, sterically hindered or electron-deficient phenoxides give monoquinolylphenols **33** and **34** as the only products. Since direct introduction of an aryl group into the **MeQone** framework is difficult, this method is considered one of the more useful modifications.

**Scheme 10:**
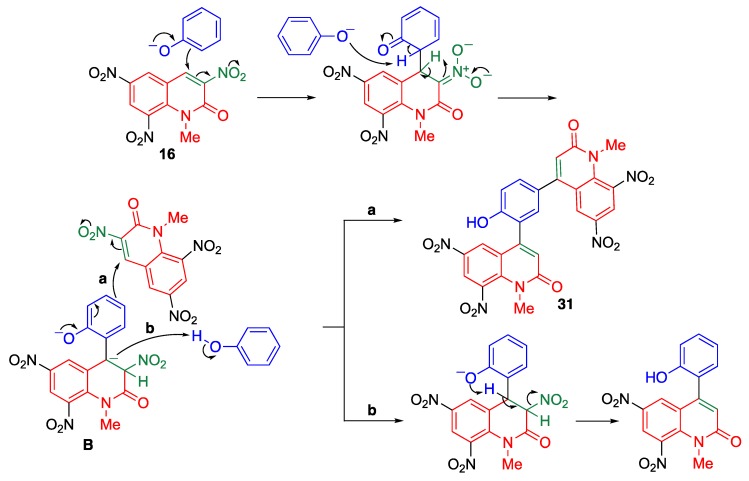
A plausible mechanism for the reaction of **16** with phenoxide.

From another viewpoint, trinitroquinolone is an aromatic compound. Hence, this reaction can be regarded as an electrophilic arylation, which is not achieved in the usual Friedel–Crafts reaction. This transformation is initiated by the nucleophilic addition of phenoxide at the 4-position of **16** (Scheme 10). The newly introduced benzene ring is aromatized with the assistance of another phenoxide. In addition, proton transfer from the 4-position to an adjacent position of the quinolone ring occurs to afford the dianionic intermediate **B**. Since **B** is a highly electron-rich species, it immediately attacks another molecule of **16** to afford bis(quinolyl)phenols **31** (path **a**). On the other hand, protonation of **B** followed by elimination of nitrous acid is the preferred route to furnish monoquinolylphenol when electron-deficient or bulky phenoxides are used (path **b**).

#### 5.1.5. *cine*-Substitution of Trinitroquinolone with Cyanides

**Scheme 11:**
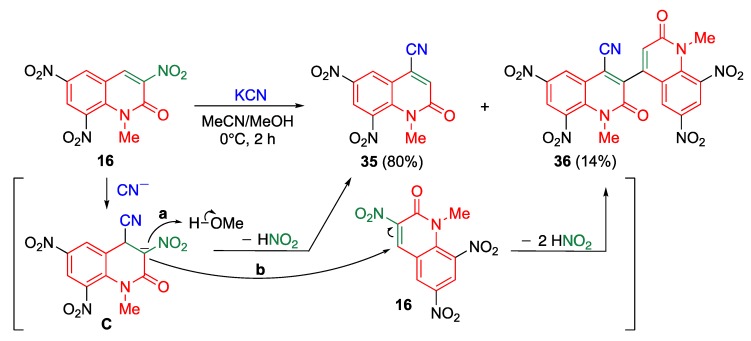
*cine*-Substitution of **16** with potassium cyanide.

Nitriles represent an important structural motif in medicinal chemistry because of their versatile biological activities [46]. In addition, they have been recognized as extremely useful intermediates for the preparation of other useful building blocks [47,48,49]. Therefore, considerable research effort has been dedicated to the development of methods for introducing cyano groups into organic molecules. Inspired by the above methods for direct C–C bond formation on the **MeQone** framework, researchers have used potassium cyanide as a carbon nucleophile for reacting with trinitroquinolone **16** to prepare 4-cyano-2-quinolone derivative **35** (Scheme 11) [42]. In this reaction, dimeric product **36** is also obtained. After the addition of a cyanide to **16**, the anionic intermediate **C** is formed, which is a common intermediate for both products **35** and **36**. When **C** is protonated, followed by the elimination of nitrous acid, **35** is obtained (path **a**). The dimeric product **36** is a result of the addition of **C** to another molecule of **16** (path **b**).

**Scheme 12:**
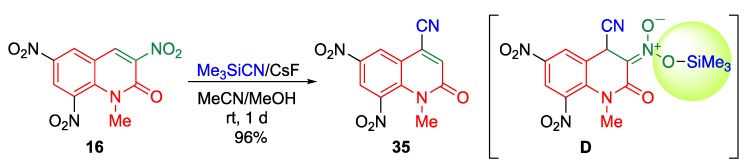
*cine*-Substitution of **16** with trimethylsilyl cyanide.

The use of trimethylsilyl cyanide/cesium fluoride instead of potassium cyanide is effective in avoiding undesired dimerization due to the steric hindrance of the *O*-silylated intermediate **D**, affording cyanoquinolone **35** as the sole product without any detectable dimer **36** (Scheme 12). While conventional strategies for cyanation of the **MeQone** framework often involve multistep reactions or harsh conditions, the present method makes the cyanation possible under mild reaction conditions with simple experimental manipulations. Thus, this protocol can be used as a powerful tool for constructing a library of versatile **MeQone** derivatives by further chemical conversion of the cyano and nitro functionalities.

The introduction of a methyl group instead of a nitro group at the 8-position also activates the **MeQone** framework. Nitrated 1,8-dimethyl-2-quinolones **37** react with potassium cyanide to afford the corresponding 4-cyano **MeQone**s (Table 11).

#### 5.1.6. Reaction of Trinitroquinolone with Tertiary Amines

As mentioned in the previous section, the cyanide ion plays two roles: it serves as a nucleophile and it stabilizes anionic intermediate because of its electron-withdrawing nature. Thus, the dimerization of **MeQone**s is also observed. Conversely, introduction of a hetero atom at the 4-position generates a stable anionic intermediate, which undergoes efficient dimerization. The treatment of trinitroquinolone **16** with a tertiary amine causes the dimerization [50]. Interestingly, more than two long alkyl chains possessing β-hydrogens are essential for undergoing this reaction (Table 12).

**Scheme 13:**
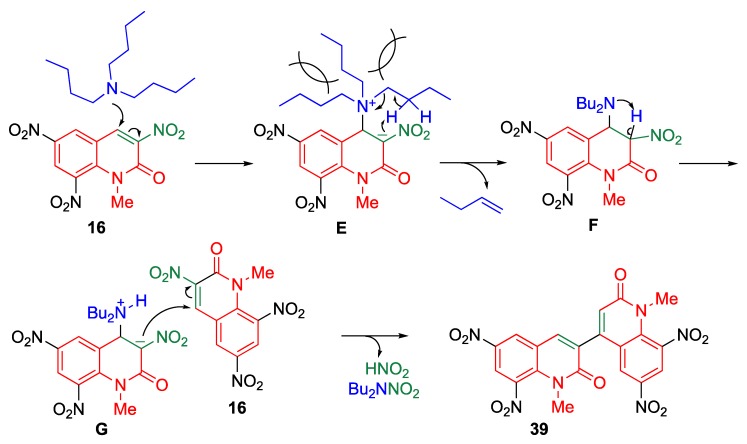
A plausible mechanism for dimerization of **16**.

This reaction is initiated by the nucleophilic addition of tributylamine to trinitroquinolone **16** to produce the zwitterion **E**. The β-elimination of 1-butene is followed by proton transfer of **F** to produce the zwitterion **G**, which reacts with another molecule of **16** to afford dimer **39** (Scheme 13).

### 5.2. Direct C–N Bond Formation at the 4-Position

#### 5.2.1. *cine*-Substitution of Trinitroquinolone with Primary Amines

**Scheme 14:**
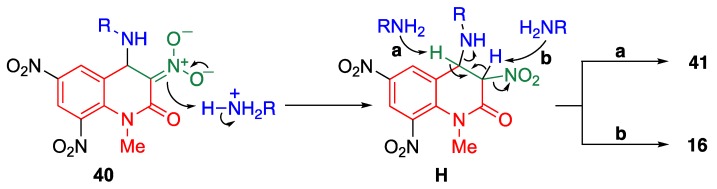
Two reaction paths leading to **41** and **16**.

A different reactivity is observed when primary amines, instead of tertiary amines, are used as the nucleophiles to react with trinitroquinolone **16**. The regioselective C–N bond formation occurs at the 4-position to afford the Meisenheimer complex **40** (Table 13) [51]. When **40** is heated, *cine*-substituted products **41a** and **41b** are obtained; however, no *cine*-substitution is observed for bulky amino substituted derivatives **40c** and **40d**, accompanied by the recovery of considerable amounts of **16**. Upon heating, **40** is converted to dihydroquinolone **H**, from which nitrous acid is eliminated to afford the *cine*-substituted products **41** (Scheme 14, path **a**). However, the elimination of amine proceeds competitively to give the trinitroquinolone **16** (path **b**), which lowers the yield of **41**.

#### 5.2.2. Amino-Halogenation and Imido-Halogenation of Quinolones

**Scheme 15:**
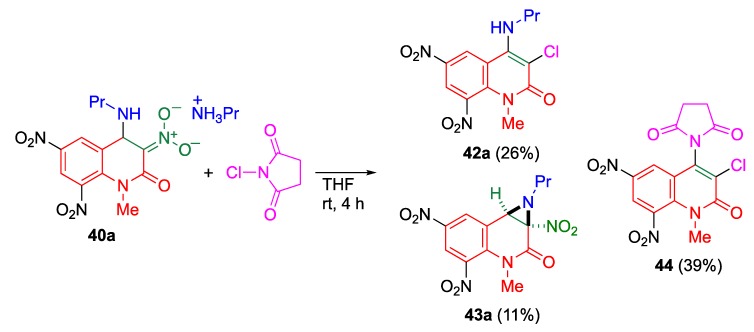
Reaction of Meisenheimer complex **40a** with NCS.

The reaction of trinitroquinolone **16** with excess propylamine in acetonitrile affords the Meisenheimer complex **40a**, which can be used for further functionalization of the **MeQone** framework upon treatment with electrophiles. When the ammonium salt **40a** is treated with *N*-chlorosuccinimide (NCS), three kinds of functionalized quinolone are obtained; the amino-chlorinated product **42**, the aziridine-fused quinolone **43**, and the imido-chlorinated product **44** (Scheme 15) [52].

**Scheme 16:**
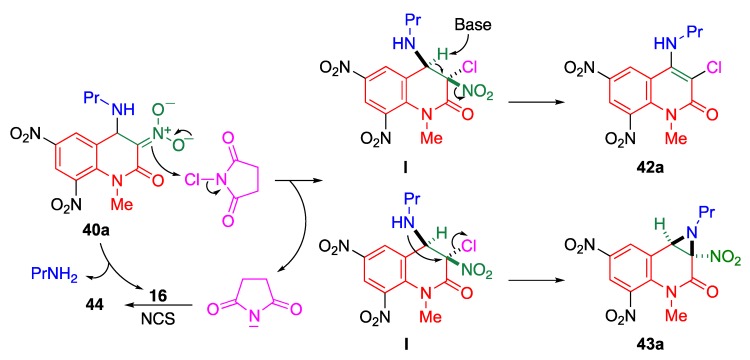
A plausible mechanism for the formation of **42a** and **43a**.

**Scheme 17:**
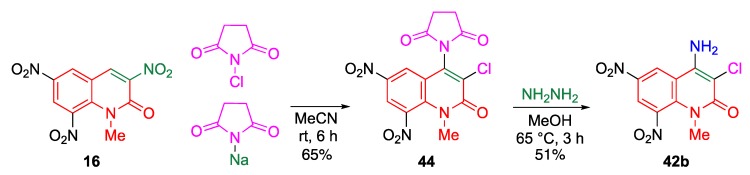
Synthesis of imido-chlorinated product **44** and the hydrazinolysis.

A plausible mechanism for these reactions is illustrated in Scheme 16. Chlorination of the Meisenheimer complex **40a** affords dihydroquinolone **I**, which is the common intermediate for **42a** and **43a**. The amino-chlorinated product **42a** is formed by elimination of nitrous acid induced by a base, such as imide anion and amine. When the amino group attacks the vicinal position to substitute chloride, an *N*-propylaziridine ring is formed to give product **43a**. On the other hand, when the eliminated imide anion reacts with trinitroquinolone **16** and NCS, the imido-chlorinated product **44** is formed, which is also formed when **16** is reacted with sodium imide in the presence of NCS (Scheme 17).

The amino-halogenation of trinitroquinolone **16** can be conducted in a one-pot two-step manner, in which the selectivity of **42** is increased by using an excess amount of amine (Table 14). The aliphatic and aromatic primary amines undergo the reaction to afford the corresponding amino-chlorinated products **42a–k** in moderate yields. However, less nucleophilic *p*-nitroaniline shows no change. While the acyclic secondary amine, diethylamine, does not furnish **42m**, the cyclic secondary amine, morpholine, yields the corresponding amino-chlorinated product **42n**. Ammonia is difficult to handle in this protocol. Instead, the imido group is considered a masked form of an amino group. Indeed, the imido-chlorinated product **44** can be transformed to the amino-chlorinated quinolone **42b** by hydrazinolysis (Scheme 17).

When NBS is employed as a halogenating reagent, a small amount of the amino-nitrated product **46** is formed in addition to the amino-brominated product **45**, presumably due to the higher leaving ability of bromide than that of chloride (Table 15). Indeed, only amino-nitrated product **46** is obtained without any detectable formation of the iodo-aminated product in the reaction with NIS.

#### 5.2.3. Aziridination of Quinolones

The screening of various 3-nitrated **MeQone**s reveals the tendency of the selectivity between amino-halogenation and aziridination (Table 16). When the electron density of the benzene ring is low, amino-chlorination occurs predominantly to afford **48a**–**c**. On the other hand, for increased electron density, intramolecular substitution exclusively occurs to form an aziridine ring, leading to **49d–g**. This tendency is considered to depend on the acidity of the proton at the 4-position in the intermediate **J**. When the acidity of H^4^ increases due to the electron-withdrawing group, elimination of a nitrous acid occurs easily via E2 reaction to give the amino-halogenated product **48**. In contrast, when the acidity of H^4^ becomes lower, an intramolecular S_N_2 reaction proceeds to afford the aziridine **49**.

**Scheme 18:**
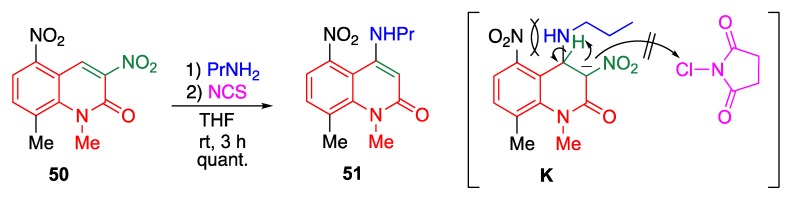
Different reactivity of 3,5-dinitro-2-quinolone **50** for the amino-chlorination.

1,8-Dimethyl-3,5-dinitro-2-quinolone **50** exhibits a reactivity different from those of the other nitroquinolones **47**. When **50** is subjected to the reaction under the same conditions, *cine*-substitution takes place, rather than amino-chlorination and aziridination, affording compound **51** quantitatively (Scheme 18). In this reaction, the addition of a propylamine affords the Meisenheimer complex **K**. However, the steric repulsion with the *peri*-substituent increases the steric hindrance around the 3-position, thus preventing the attack to NCS. Instead, proton transfer from the 4-position followed by elimination of the nitrite ion affords the *cine*-substituted product **51**.

**Scheme 19:**
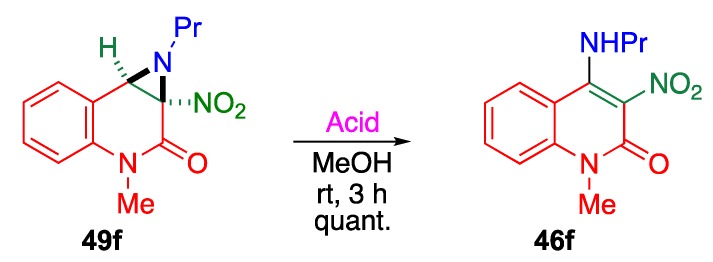
Aziridine ring opening leading to vicinally functionalized quinolone.

Aziridine-fused quinolone **49f** undergoes a ring-opening reaction followed by rearomatization upon treatment with acid, such as toluenesulfonic acid, hydrochloric acid, and trifluoroborane, to furnish the amino-nitrated **MeQone** (Scheme 19).

### 5.3. Direct C–O Bond Formation at the 4-Position

When trinitroquinolone **16** is treated with a sodium alkoxide at room temperature, nucleophilic addition at the 4-position affords an alkoxylated salt **52** [53], which can be isolated because of stabilization by the adjacent nitro and carbonyl groups. After removal of alcohol, treatment of the adduct **52** with NCS in acetonitrile affords the 4-alkoxy-3-chloro-2-quinolone derivatives **53** in moderate-to-high yields (Table 17). This protocol can be performed in a one-pot manner with simple experimental manipulations.

**Scheme 20:**
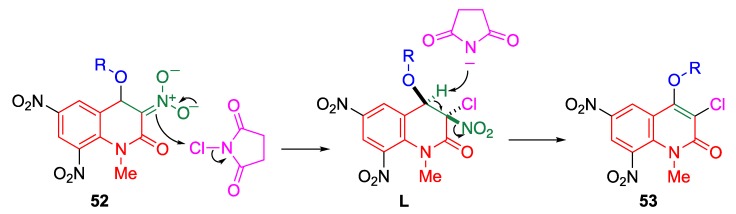
A plausible mechanism for alkoxy-chlorination of **MeQone**s.

The reaction proceeds via a similar mechanism, as shown in Scheme 16, for the amino-chlorination (Scheme 20). Chlorination of the alkoxylated salt **52** with NCS affords the dihydroquinolone intermediate **L**, from which nitrous acid is eliminated to form a bis(functionalized) product **53**.

When NBS is used as the halogenating reagent, 4-methoxylated trinitroquinolone **54** is obtained in addition to the methoxy-brominated product **53** (Table 18). In the reaction using NIS, product **54** is furnished without any detectable formation of **53**. The different reactivity is due to the higher leaving abilities of bromide and iodide than that of chloride.

**Scheme 21:**
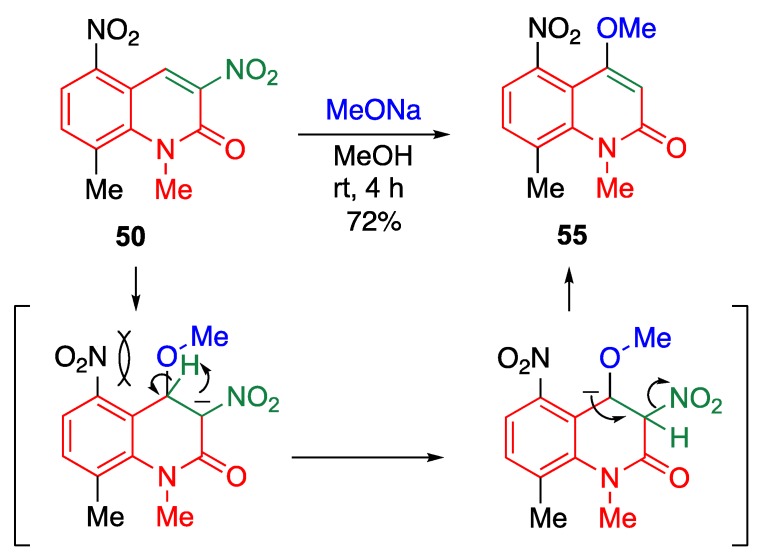
*cine*-Substitution of 3,5-dinitro-2-quinolone **50** by sodium methoxide.

3,5-Dinitro-2-quinolone **50** exhibits a reactivity similar to that observed in amino-chlorination to afford the *cine*-substituted product **55** (Scheme 21). Although the addition of methoxide to **50** occurs at the 4-position, it cannot react with NCS at all because of steric repulsion between the 4-methoxy and 5-nitro groups. Instead, proton transfer followed by elimination of nitrite anion affords the *cine*-substituted product **55**.

## 6. Conclusions

In this review, recent progress in the nitro-promoted direct functionalization of pyridones and quinolones was summarized. A variety of functionalities can be easily introduced into pyridone and quinolone frameworks via activation of the nitro group, facilitating the preparation of newly functionalized derivatives. These methods can promote the construction of a library of pyridones and quinolones with potentially interesting and valuable bioactivities. It is expected that more intensive research in this exciting field will establish the nitro-promoted direct functionalization of heterocycles as a powerful and broadly applicable synthetic strategy in organic synthesis.

## Figures and Tables

**Table 1:**
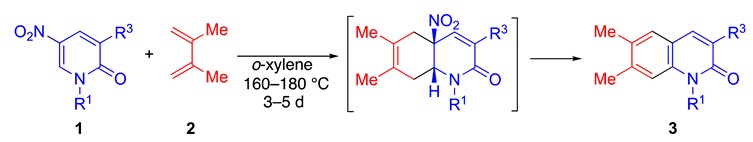
D-A cycloaddition of 5-nitropyridones **1** with diene **2**.

R^1^	R^3^		Yield/%
H	H	**a**	26
Me	H	**b**	30
Me	COOMe	**c**	22

**Table 2:**
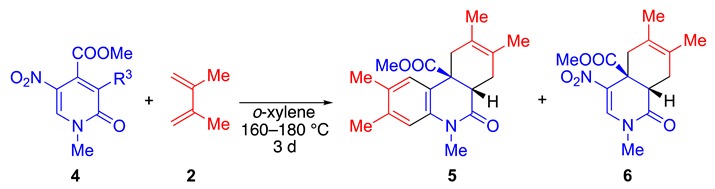
D-A reaction of 5-nitropyridones **4** accompanying elimination of the nitro group.

R^3^		Yield/%	
		5	6
H	**a**	27	10
NO_2_	**b**	33	15

**Table 3:**
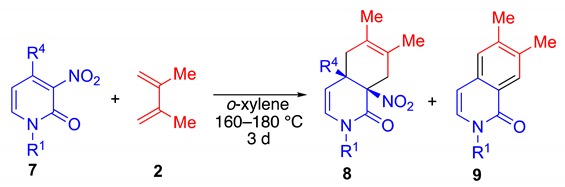
Cycloaddition of 3-nitropyridones **7** with diene **2**.

R^1^	R^4^		Yield/%	
			8	9
H	H	**a**	0	15
Me	H	**b**	20	22
Me	COOMe	**c**	36	0

**Table 4:**
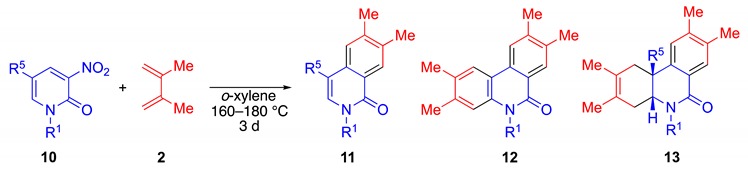
Cycloaddition of 5-substituted 3-nitropyridones **10** with diene **2**.

R^1^	R^5^		Yield/%		
			11	12	13
H	NO_2_	**a**	13	15	0
Me	NO_2_	**b** ^1^	36	33	0
Me	COOMe	**c**	31	0	14
H	COOMe	**d**	13	0	5

^1^ 8% of **8b** is obtained.

**Table 5:**
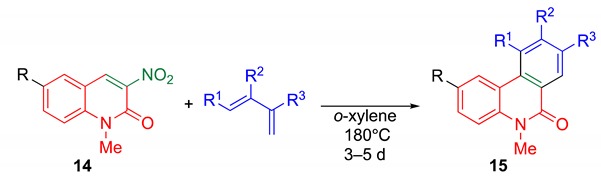
Cycloaddition of 3-nitrated quinolones **14** with dienes **2**.

R	R^1^	R^2^	R^3^		Yield/%
H	OMe	H	H	**a**	83
NO_2_	OMe	H	H	**b**	68
H	H	Me	Me	**c**	95
NO_2_	H	Me	Me	**d**	64
H	H	OMe	OMe	**e**	45
NO_2_	H	OMe	OMe	**f**	13

**Table 6:**
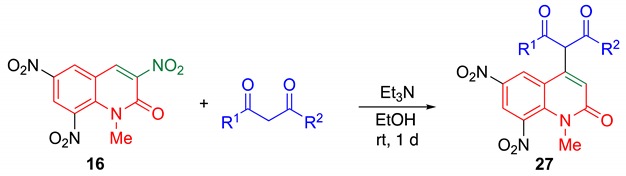
*cine*-Substitution of **16** with 1,3-dicarbonyl compounds.

R^1^	R^2^		Yield/%
Me	Me	**a**	88
-(CH_2_)_3_-		**b**	68
Me	OEt	**c**	93
CH_2_COOEt	OEt	**d**	26
OEt	OEt	**e**	93

**Table 7:**
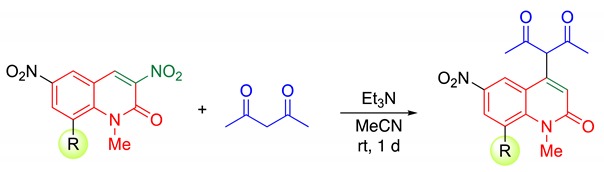
Effect of the substituent at the 8-position for the *cine*-substitution.

R	Yield/%
NO_2_	88
H	0 ^1^
Me	92

^1^ At 80 °C.

**Table 8:**
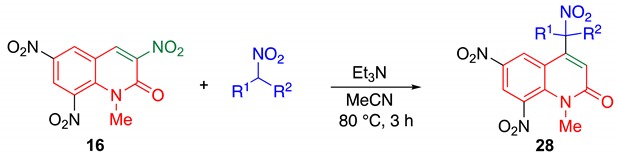
*cine*-Substitution of **16** with nitroalkanes.

R^1^	R^2^	Yield/%
Me	H	80
Et	H	98
Me	Me	77 ^1^

^1^ For 1 d.

**Table 9:**
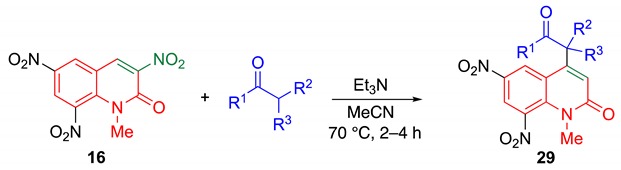
*cine*-Substitution of **16** with ketones.

R^1^	R^2^	R^3^	Yield/%
H	Me	Me	41
Me	H	H	83
Ph	H	H	83
Et	Me	H	18
-(CH_2_)_4_-		H	82
Ph	Me	H	77
Ph	Ph	H	69
2-furyl	H	H	45
2-pyridyl	H	H	74

**Table 10:**
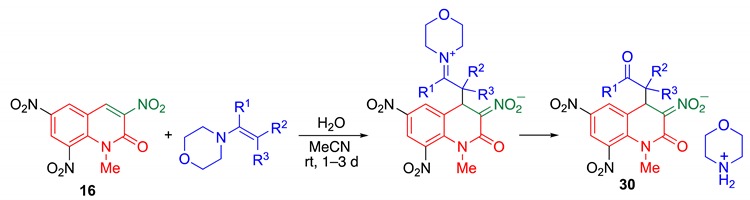
*cine*-Substitution of **16** with enamines.

R^1^	R^2^	R^3^	Yield/%
H	Me	Me	98
-(CH_2_)_4_-		H	40
Ph	Me	H	43
Ph	Ph	H	98

**Table 11:**
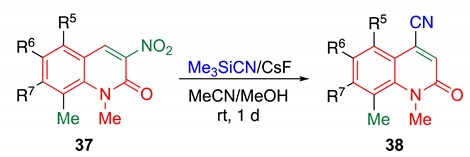
*cine*-Substitution of nitrated 1,8-dimethyl-2-quinolones with trimethylsilyl cyanide.

R^5^	R^6^	R^7^		Yield/%
NO_2_	H	NO_2_	**a**	83
NO_2_	H	H	**b**	47
H	NO_2_	H	**c**	quant.

**Table 12:**
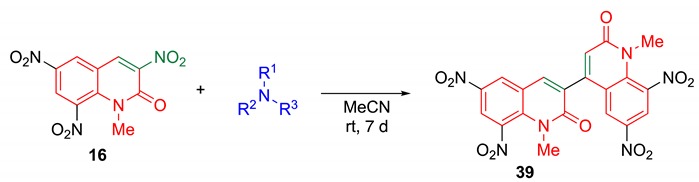
Reactions of **16** with tertiary amines.

R^1^	R^2^	R^3^	Yield/%
Me	Me	Me	0
Et	Et	Et	34
Pr	Pr	Pr	76
Bu	Bu	Bu	93
Bu	Bu	Me	79
Bu	Me	Me	18
PhCH_2_	PhCH_2_	PhCH_2_	0

**Table 13:**
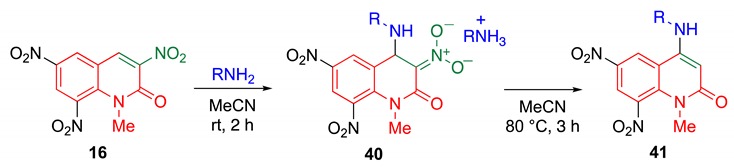
*cine*-Substitution of **16** with primary amines.

R		Yield/%	
		40	41
Pr	**a**	71	36
*i*-Bu	**b**	74	29
*s*-Bu	**c**	56	0
*t*-Bu	**d**	74	0

**Table 14:**
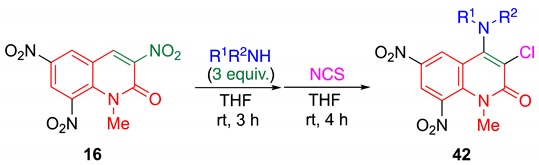
One-pot amino-chlorination of trinitroquinolone **16**.

R^1^	R^2^		Yield/%
Pr	H	**a**	62
*i*-Bu	H	**c**	70
*s*-Bu	H	**d**	49
PhCH_2_	H	**e**	54
HOCH_2_CH_2_	H	**f**	56
CH_2_=CHCH_2_	H	**g**	35
Ph	H	**h**	54
4-MeOC_6_H_4_	H	**i**	37
4-BuC_6_H_4_	H	**j**	41
4-IC_6_H_4_	H	**k**	62
4-NO_2_C_6_H_4_	H	**l**	trace
Et	Et	**m**	0
-(CH_2_)_2_-O-(CH_2_)_2_-		**n**	62

**Table 15:**
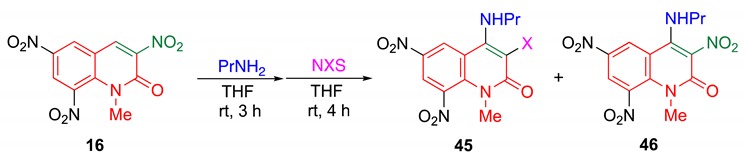
Scanning of halogenating agents.

X	Yield/%	
	45	46
Cl	62	0
Br	63	16
I	0	62

**Table 16:**
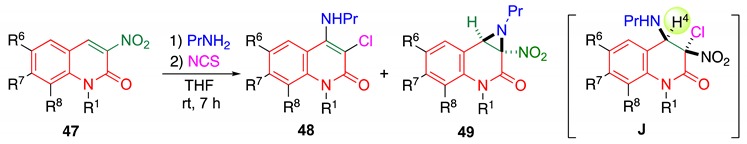
Amino-chlorination and aziridination of various 3-nitrated **MeQone**s.

R^1^	R^6^	R^7^	R^8^		Yield/%	
					48	49
Me	NO_2_	H	NO_2_	**a**	62	0
Me	NO_2_	H	Me	**b**	13	21
Me	NO_2_	H	H	**c**	13	49
Me	Br	H	H	**d**	trace	68
Me	H	H	H	**e**	0	65
Me	H	H	H	**f**	0	71
H	H	H	H	**g**	0	61

**Table 17:**
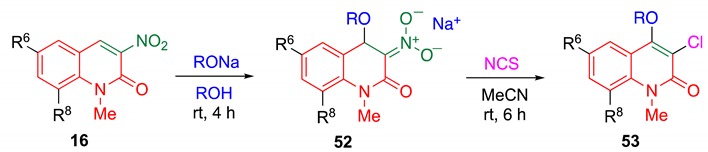
Alkoxy-chlorination of **16** leading to **53**.

R	R^6^	R^8^		Yield/%	
				52	53
Me	NO_2_	NO_2_	**a**	81	85
Et	NO_2_	NO_2_	**b**	quant.	73
*i*-Bu	NO_2_	NO_2_	**c**	-	46
*i*-Pr	NO_2_	NO_2_	**d**	-	45
PhCH_2_CH_2_	NO_2_	NO_2_	**e**	-	55
CH_2_=CHCH_2_	NO_2_	NO_2_	**f**	-	51
HC≡CCH_2_	NO_2_	NO_2_	**g**	-	29
Me	NO_2_	Me	**h**	-	78
Me	NO_2_	H	**i**	-	73
Me	H	H	**j**	-	65

**Table 18:**
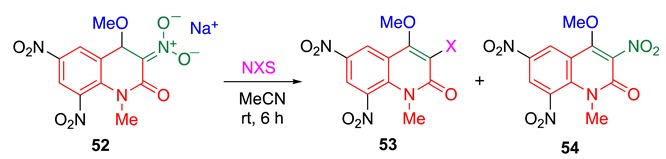
Scanning of halogenating agents.

X	Yield/%	
	53	54
Cl	85	0
Br	62	27
I	0	29
